# Editorial: Mechanisms and Therapy for Cancer Metastasis to the Central Nervous System

**DOI:** 10.3389/fonc.2019.00064

**Published:** 2019-02-12

**Authors:** Haotian Zhao, David D. Eisenstat

**Affiliations:** ^1^Department of Biomedical Sciences, New York Institute of Technology College of Osteopathic Medicine, New York, NY, United States; ^2^Departments of Oncology, Medical Genetics and Pediatrics, Faculty of Medicine & Dentistry, University of Alberta, Edmonton, AB, Canada

**Keywords:** driver mutation, macrophage microglia activation, reactive astrocyte, xenografts model, cerebrospinal fluid – CSF, brain metastases

Metastasis of malignancies to the brain, including parenchymal and leptomeningeal disease, represents a common neurological complication in cancer patients (1, 2). Currently, ~10% of all cancer patients experience involvement of the central nervous system (CNS) (3, 4). Though ~40% of patients with metastatic cancers are affected by brain metastases (1, 5), commonly used treatment options for brain metastases such as surgery or radiotherapy are associated with only modest benefits (6).

The study of tumor cell metastasis to the brain or leptomeninges has developed into an intensely researched area, as it has the potential to provide selective targets for developing therapeutic strategies for brain metastases. In this Research Topic, we have organized a collection of review and original research articles that will hopefully help the readers gain insight into different aspects of cancer metastases to the CNS. In an overview of brain metastasis, Franchino et al. describe different detection methods, including magnetic resonance imaging (MRI), computed tomography, positron emission tomography (PET) as well as advanced imaging techniques commonly used in the diagnosis, treatment planning, and follow-up of patients with brain metastases. The authors also introduce more sophisticated methods of tumor analysis to detect circulating biomarkers in body fluids such as blood and cerebrospinal fluid, and their use in monitoring treatment response and tumor progression. The benefits and impacts on prognosis of different commonly used therapeutic approaches are discussed, including more recent clinical trials featuring immunotherapies such as checkpoint inhibitors.

In the era of precision medicine, choice of cancer treatment has been increasingly prescribed based on the molecular or genomic properties of the individual cancer. Han and Brastianos describe novel approaches in genomic testing such as the use of cell-free circulating tumor DNA in the cerebrospinal fluid in the study of brain metastases. The authors focus on recent advances in genomic profiling of brain metastases and current knowledge of targeted therapies in the management of brain metastases from cancers of the breast, lung, colorectum, kidneys, and ovaries as well as melanoma. Identification of genomic alterations found in brain metastases and targeted therapies against these mutations represent an important research area that could potentially bring improved outcomes for patients with brain metastases.

Brain metastasis and leptomeningeal spread are process that can be partially recapitulated through *in vitro* assays. Despite their extensive use, these assays have limitations in the study of complex host/tumor cell interactions throughout metastasis. In this respect, animal models represent a versatile tool to examine anatomical barriers (such as the blood brain barrier), stromal/environmental factors, genetic factors, and the immune response. Singh et al. discuss the development of patient-derived xenograft (PDX) models for brain and leptomeningeal metastasis through injection of patient tumor cells into an appropriate microenvironment. These PDX models have been shown to maintain their molecular signatures and recapitulate the heterogeneous nature of the original patient tumor, providing some advantages over genetically engineered mouse models. These models have contributed significantly to our understanding of CNS metastasis, and will play a pivotal role in the identification and testing of potential therapeutic agents.

Despite improvements in systemic therapies, brain metastasis of breast cancer is associated with dismal survival with limited and non-specific treatment options. Study of breast cancer brain metastases and preclinical tests have relied mostly on injection of a few breast cancer cell lines; however, this approach suffers from a lack of tumor heterogeneity observed clinically and limited use for therapeutic studies due to their rapid progression following transplantation. Contreras-Zárate et al. developed and characterized a PDX model of breast cancer brain metastasis. The investigators obtained freshly resected brain metastases from breast cancer and implanted tumor cells in the mammary fat pad of female immune-deficient recipients. The xenografts retained critical clinical markers and gene expression profiles of parental tumors. Importantly, intra-cardiac injection of dissociated cells from xenografts led to tumors in the brain parenchyma within 8–12 weeks, suggesting that xenografts derived from brain metastases maintain the capacity to colonize the brain. These novel xenografts represent heterogeneous and clinically relevant models to study the biology of brain metastasis and to test drugs in therapeutically relevant settings.

Indeed, PDX models recapitulate many characteristics of breast cancer brain metastases including active angiogenesis and astroglial activation. Conversely, unlike other sites for tumor dissemination such as liver or bone, astrocytes represent a major cell type that come into contact with cancer cells during brain metastasis. In response to cancer cells that metastasize to the brain, astrocytes undergo further differentiation and activation to affect the survival and growth of disseminated cancer cells within the CNS. Wasilewski et al. discuss our current understanding of the contribution of reactive astrocytes to brain metastasis. Emphasis is placed on the signaling pathways and interactions that play a crucial part in the communication with metastatic cancer cells (7). In addition to astrocytes, local macrophages and microglia in the CNS constitute important components of the immune response to metastatic growth. Accumulation of microglial cells surrounding metastatic tumors has been described for both experimental and human brain metastases. Andreou et al. examined microglial/macrophage activation in a mouse model of breast cancer brain metastasis. Microglia can differentiate along the proinflammatory pathway to upregulate cytokine levels and acquire the ability to mediate an immune response against tumor cells. Alternatively, microglia can develop an anti-inflammatory phenotype that promotes angiogenesis and tumor growth. The authors identified populations of both proinflammatory and anti-inflammatory microglia/macrophages. The investigators further demonstrated that selective depletion of this microglia/macrophage population significantly reduced metastatic tumor burden and increased apoptosis. These findings suggest that microglia/macrophages are important effectors of the inflammatory response in brain metastases. Hence targeting the anti-inflammatory pathway in microglia/macrophages may offer therapeutic opportunities for patients with brain metastases.

Treatment options for intracerebral seeding of cancer cells are limited and lacking specificity. The molecular and cellular makeup of brain metastases usually differs from that of the primary tumors, as well as from metastases at other sites. Molecular detailing of metastatic cancer cell penetration, seeding, and outgrowth in the brain will contribute to the discovery of innovative cancer therapies.

## Author Contributions

All authors listed have made a substantial, direct and intellectual contribution to the work, and approved it for publication.

### Conflict of Interest Statement

The authors declare that the research was conducted in the absence of any commercial or financial relationships that could be construed as a potential conflict of interest.
